# Characterizing post-branching nephrogenesis in the neonatal rabbit

**DOI:** 10.1038/s41598-023-46624-9

**Published:** 2023-11-06

**Authors:** Meredith P. Schuh, Sunitha Yarlagadda, Lyan Alkhudairy, Kristina Preusse, Raphael Kopan

**Affiliations:** 1 Department of Pediatrics, University of Cincinnati College of Medicine, Cincinnati, OH USA; 2 Division of Nephrology and Hypertension, Cincinnati Children’s Hospital Medical Center, 3333 Burnet Ave, MLC 7022, Cincinnati, OH 45229 USA; 3 Division of Developmental Biology, Cincinnati Children’s Hospital Medical Center, Cincinnati, OH USA

**Keywords:** Developmental biology, Morphogenesis, Kidney, Nephrons

## Abstract

Human nephrogenesis ends prior to birth in term infants (34–36 week gestation), with most (60%) nephrons forming in late gestation in two post-branching nephrogenesis (PBN) periods: arcading and lateral branch nephrogenesis. Preterm infants, however, must execute PBN postnatally. Extreme prematurity is associated with low nephron counts. Identifying additional model(s) that undergo PBN postnatally will help support postnatal PBN in preterm infants. The rabbit exhibits longer postnatal nephrogenesis than the mouse but whether it forms nephrons through PBN has not been determined. We performed morphologic and immunohistological assessments of rabbit nephrogenesis from birth (post-conceptual day 31 or 32) to PC49 using H&E and antibodies against SIX1, SIX2, WT1, ZO-1, and JAG1 in the postnatal period. We performed 3D rendering of the nephrogenic niche to assess for PBN, and supplemented the staining with RNAScope to map the expression of *Six1*, *Six2* (nephron progenitors, NPC), and *Ret* (ureteric bud tip) transcripts to determine the nephrogenic niche postnatal lifespan. Unlike the mouse, rabbit SIX2 disappeared from NPC before SIX1, resembling the human niche. Active nephrogenesis as defined by the presence of SIX1 + naïve NPC/tip population persisted only until PC35–36 (3–5 postnatal days). 3D morphologic assessments of the cortical nephrons identified an elongated tubule with attached glomeruli extending below the UB tip, consistent with PBN arcades, but not with lateral branch nephrogenesis. We conclude that the rabbit shows morphologic and molecular evidence of PBN arcades continuing postnatally for a shorter period than previously thought. The rabbit is the first non-primate expressing SIX1 in the progenitor population. Our findings suggest that studies of arcading in postnatal nephrogenic niche should be performed within the first 5 days of life in the rabbit.

## Introduction

Human nephron number (nephron endowment) ranges from 0.2 to 2.7 million nephrons per kidney^1–3^. Human nephrogenesis is complete prior to birth at 34–36 weeks gestation with 60% of nephrons formed during third trimester of development^4^. Infants born preterm must attempt to complete nephrogenesis postnatally but may undergo abbreviated and/or abnormal nephrogenesis ex-utero^5,6^. Disruption of this critical period of nephrogenesis by preterm birth is deleterious: preterm infants are at the low end of nephron endowment and at high risk for chronic kidney disease (CKD) and end stage kidney disease (ESKD) later in life^7–9^. Thus, identifying preclinical models that simulate human postnatal nephrogenesis in the preterm infant with a prolonged window amendable to manipulation is required to gain an understanding of nephrogenesis in infants born preterm and, ultimately, to interventions.

Vertebrate nephrogenesis is driven by reiterative interaction between the mesenchymal nephron progenitor cells (NPC) and the epithelial ureteric tip. To form a nephron, a group of NPC undergo mesenchymal to epithelial transition (MET) at stereotyped positions^10,11^. Nephrogenesis proceeds in at most three phases. First, the UB undergoes branching morphogenesis, and each branch generates between two and three nephrons. We will refer to this as branching phase nephrogenesis (BpN)^12^; mice generate the majority of their nephrons at this phase^13^. BpN produces ~ 33,000–42,000 nephrons (2^15^)^14^ in humans.

BpN is followed by two post-branching nephrogenesis (PBN) phases in the human. The first post-branching phase is known as arcading, observed between 15 and 22 weeks gestation in humans^15^. Arcades are morphologically characterized by immature nephrons directly connected to each other in chains that drain into a single collecting tubule^14,15^. During arcading, the UB does not branch nor does it elongate significantly. In the mouse between postnatal day 2 (P2) and P3, there is en masse differentiation of the progenitor pool forming multiple nephrons attaching to a single tip and nephrogenesis is completed^16,17^. While some may see this as an amplifying process analogous to arcading, the nephrons do not form an arcade by connecting to each other. In the human (and other primates^18^), there is one final phase known as lateral branch nephrogenesis (LBN) that characterizes nephrogenesis from 22 weeks gestation onwards. LBN is typified by an elongating UB stalk with an active UB tip that induces nephrons periodically, each directly connecting to the ureteric stalk as it undergoes MET^14,15,19^. PBN in the human kidney must create 15–75 nephrons at or near each of the 33,000–42,000 terminal branch tips to bring the final nephron count from 200,000 or 2.7 million, respectively^14,20^. It is the phase most affected by prematurity.

Herein we characterize the rabbit postnatal nephrogenesis. Earlier studies in the rabbit^21^ identified morphologic evidence for both BpN and arcading within the kidney cortex, and reported that postnatal nephrogenesis continued up to 10–14 days^22,23^ enabling study and manipulation of late nephrogenesis *ex-utero*. Indeed, application of indomethacin and gentamicin after 1 week of life to model acute kidney injury reduced nephron numbers^24^. However, the duration of the arcading phase is unclear, as is the question of whether rabbit postnatal nephrogenesis concludes in arcading or undergoes a limited LBN phase. To address these questions, we performed molecular studies of the postnatal nephrogenic niche in the rabbit.

We report that while the rabbit nephrogenic zone is still detected for 10–11 days postnatally, a niche containing naïve NPC population and UB tip disappears as early as 4–5 days postnatally. Like primates, the rabbit exhibits rosette-like orientation of asymmetric nephrogenic niches, and 3D morphologic assessments identified an elongated tubule with attached glomeruli extending below the UB tip, consistent with arcades. We found no evidence of LBN. Based on this molecular assessment, the rabbit PBN period consists exclusively of arcading, and although shorter than what was assumed based on morphological studies, could serve as a tractable model to study the postnatal development in the extremely preterm infant.

## Results

### Molecular assessment of rabbit nephrogenesis shows early exit of nephrogenic niche

**Figure 1:**
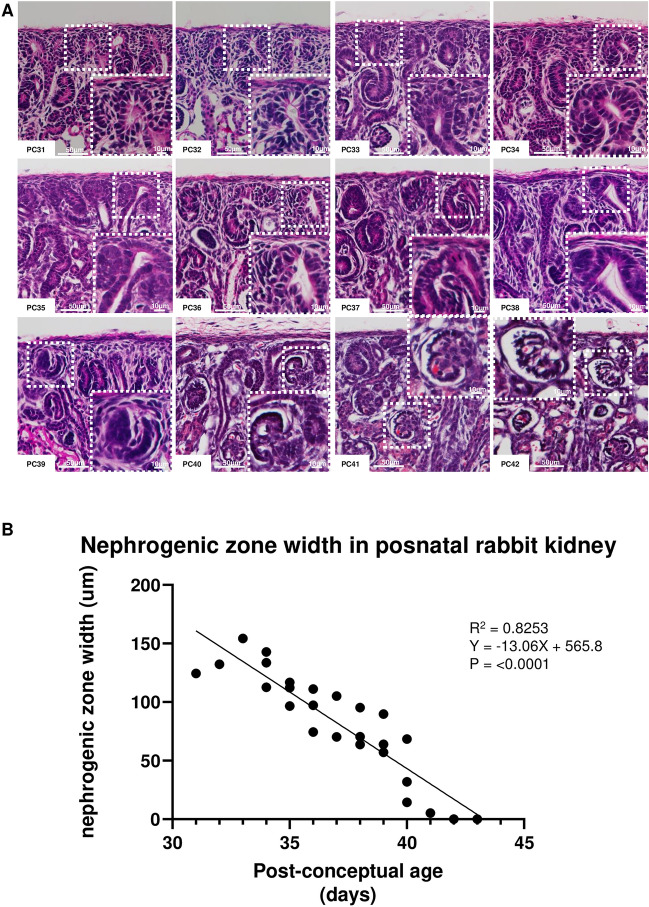
Nephrogenic zone visible until post-conceptual day 40–41 (PC40-41). (**a**) We identified a dense, tightly packed mesenchyme above the ureteric bud (UB) as the cap mesenchyme (inset) which persisted until PC36. Immature nephrons were present until PC39. By PC40-41, only mature glomeruli could be seen. (**b**) Linear regression analysis of gestational age versus nephrogenic zone width in rabbits from PC31-PC42.

In order to characterize postnatal nephrogenesis in the rabbit, we analyzed rabbit nephrogenesis from birth (post-conceptual age; PC31–PC32) to PC49 (after which we see no meaningful changes beyond collecting system elongation). As date of birth ranged from PC31-PC32 for the litters, we maintained the post conceptual naming schema for consistency. However, postnatal age (P) is also listed for clarity. Hematoxylin and eosin (H&E) staining was performed to histologically examine the progression of postnatal kidney development during the first 2–3 weeks of life (Fig. 1A). A dense, tightly packed mesenchyme persisted until PC37 (P5–P6) capping the UB asymmetrically (inset), where NPC may be present. Immature nephrons were present until PC39–PC40 (P7–P9). By PC40–PC41 (P8–P10), only mature glomeruli could be seen. As a previously validated method of determining ongoing nephrogenesis^6,25,26^, we also measured the nephrogenic zone (NZ) width, and determined that the NZ was present until PC40–PC41 (P8–P10), consistent with previous literature of nephrogenesis continuing roughly 10 days after birth (Fig. 1B).

**Figure 2:**
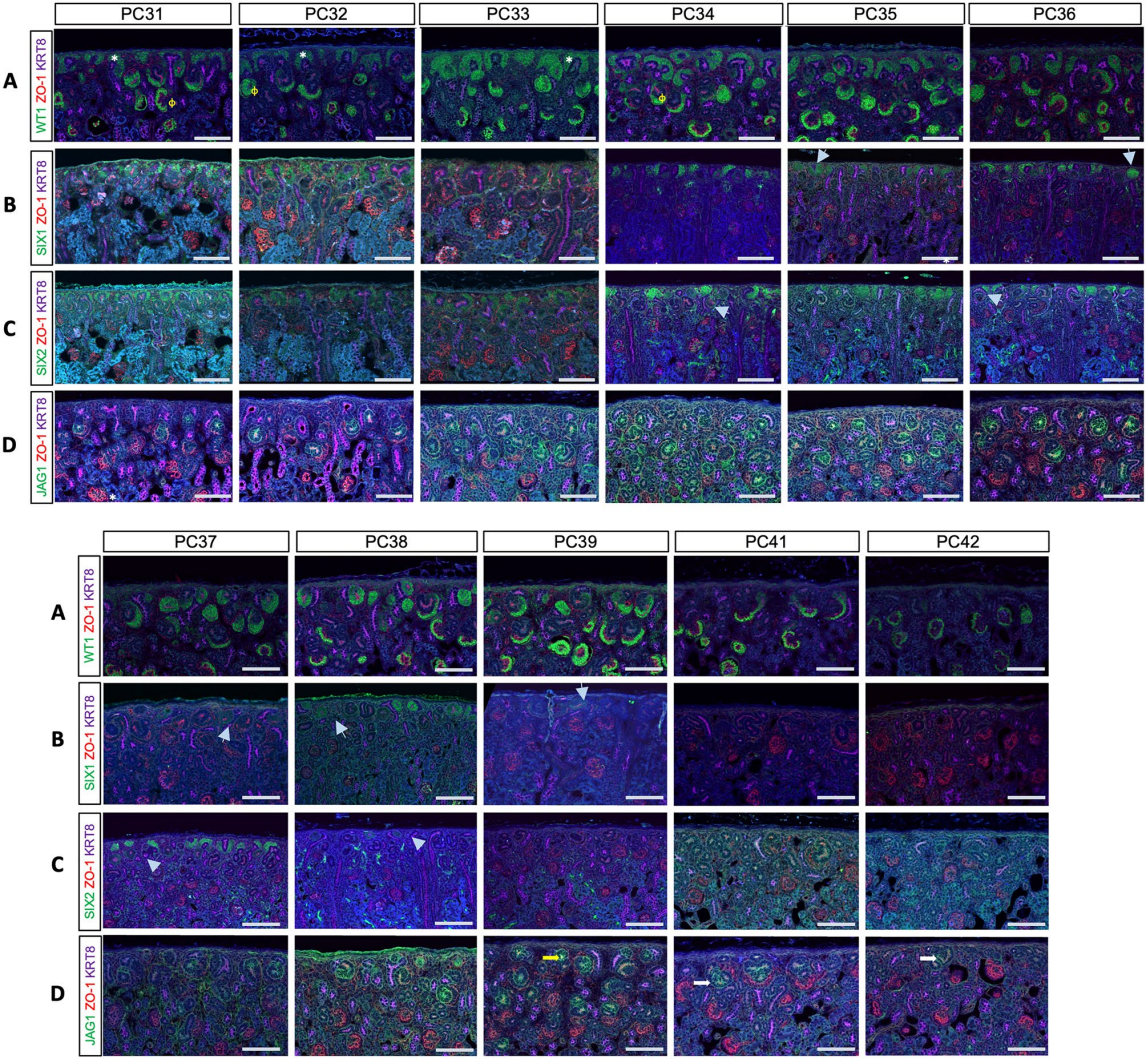
Molecular assessment of nephrogenesis cessation demonstrates undifferentiated NPCs until PC35. Rabbit samples were immunostained for WT1 (**A**), SIX1 (**B**), SIX2 (**C**), and JAG 1 (**D**), along with KRT8/18 (purple) and ZO-1 (red). Gray arrows identify renal vesicles (RV) staining with SIX1 or SIX2. White stars identify WT1+ NPC populations without ZO-1 that have not yet epithelialized and therefore represent naïve NPC, while ɸ represents mature WT1 staining in the glomerular podocytes. SIX2 is seen in differentiated NPC until PC37-38, while SIX1 persists until PC39-40. JAG1 (**D**) is visualized until PC40-41. Scale bar = 100 microns.

We next set out to assess the persistence of NPC by detecting the transcription factors (TFs) SIX1 and SIX2. SIX2 is essential for NPC self-renewal and maintenance. Unlike the mouse NPC which only express SIX2, the human NPC express both SIX1 and SIX2 throughout active nephrogenesis^27^. The rabbit resembles the human with both SIX1 and SIX2 persisting in the early differentiating nephrons (renal vesicles (RV; gray arrows, Fig. 2B,C). To identify NPC undergoing MET^28^, we performed immunofluorescence staining to detect the epithelial tight junction protein ZO-1^29^. Three animals were analyzed at each time point from PC33-40, with representative images shown in Fig. 2. SIX2 + /ZO-1- and SIX1 + /ZO- NPC persisted until PC35 in two out of three rabbit kits and until PC36 in one out of three rabbit kits. However, by PC36–PC37 (P4-6) all SIX2 + underwent MET and became ZO-1 + . Unlike SIX2, SIX1 + /ZO-1 + NPC were detected up to PC40 (P8-9) in one out the three animals examined at this age, suggesting that SIX1 expression may persist longer than SIX2, resembling the human^27^. WT1 expression marking NPC and developing podocytes was detected in the NPC no later than PC36 (P4-5) before becoming restricted to the epithelia (Fig. 2A). This multi-marker assessment supports depletion of the naïve NPC pool early in postnatal development by 4–5 days of life. Details on the expression data for each rabbit analyzed can be seen in Table 1.

**Table 1:** Nephrogenic scoring system of ongoing nephrogenesis in the postnatal rabbit. One point awarded for any SIX1, pure SIX1 (without epithelialization/co-staining with ZO-1), any SIX2, pure SIX2 (without epithelialization/co-staining with ZO-1), and WT1 without ZO-1 in the niche tip. Score of 5 equates to ongoing nephrogenesis while a score of = 0 lacks any evidence of ongoing nephrogenesis. Naïve NPC (without ZO-1 co-staining) are depleted by PC35-36.

Post-conceptual Age	Six1 (any)	Six1 (without zo-1)	Six2 (any)	Six2 (without zo-1)	WT1 (without zo-1)	Nephrogenesis score
PC 31	Yes	Yes	Yes	Yes	Yes	5
PC 32	Yes	Yes	Yes	Yes	Yes	5
PC 33	Yes	Yes	Yes	Yes	Yes	5
PC 34 #1	Yes	Yes	Yes	Yes	Yes	5
PC 34 #2	Yes	Yes	Yes	Yes	Yes	5
PC 34 #3	Yes	Yes	Yes	Yes	Yes	5
PC 35 #1	Yes	Yes	Yes	Yes	Yes	5
PC 35 #2	Yes	No	Yes	No	No	2
PC 35 #3	Yes	Yes	Yes	Yes	Yes	5
PC 36 #1	Yes	Yes	Yes	Yes	Yes	5
PC 36 #2	Yes	No	Yes	No	No	2
PC 36 #3	Yes	No	Yes	No	No	2
PC 37 #1	Yes	No	Yes	No	No	2
PC 37 #2	Yes	No	Yes	No	No	2
PC 38 #1	Yes	No	No	No	No	1
PC 38 #2	Yes	No	Yes	No	No	2
PC 38 #3	Yes	No	Yes	No	No	2
PC 39 #1	Yes	No	No	No	No	1
PC 39 #2	No	No	No	No	No	0
PC 39 #3	Yes	No	No	No	No	1
PC 40 #1	No	No	No	No	No	0
PC 40 #2	No	No	No	No	No	0
PC 40 #3	Yes	No	No	No	No	1

**Figure 3:**
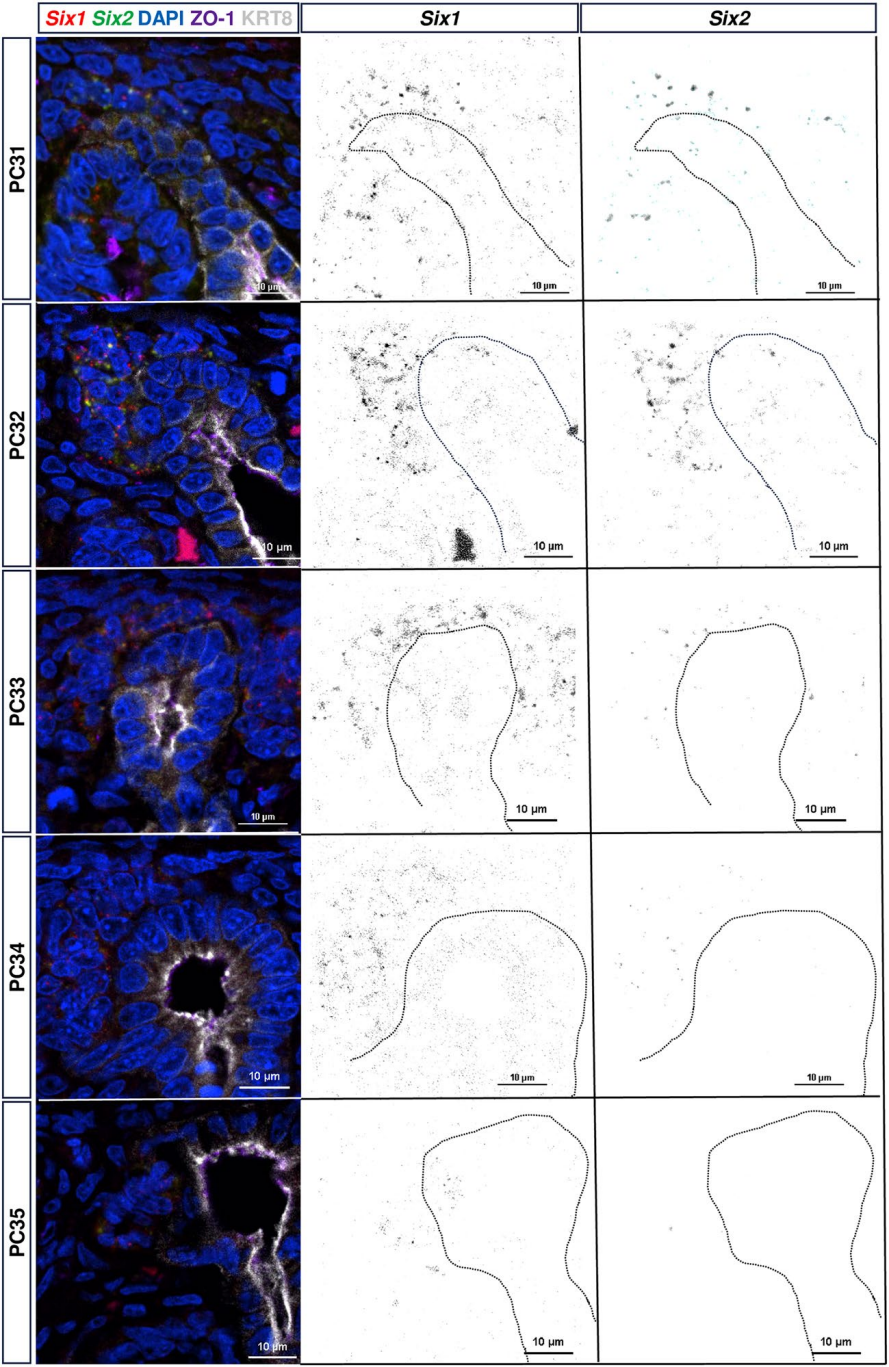
Multiplex In Situ Hybridization confirms co-expression of *Six1* and *Six2* until PC34, with persistence of *Six1* + transcripts in the nephrogenic niche. Images obtained at 100 × magnification show transcripts expressed within the same cell population of the nephrogenic niche. Despite protein expression until PC36, early *Six2* exit from the niche is consistent with human literature supporting persistent *SIX1* expression rather than *SIX2 *expression in the nephron progenitor population.

To ask if SIX1/2 were co-expressed, we used multiplex RNA in situ hybridization (ISH) (RNAScope©) on kidneys isolated from PC31 to PC35 (Fig. 3, supplemental Fig. 1). In addition to confirming co-expression of *Six1* and *Six2* transcripts within the same NPC until PC33, we confirmed that *Six2* transcripts were greatly diminished by PC34 and no longer visible in the niche at PC35 while *Six1* transcripts persisted.

We next assessed nascent nephron formation by detecting the Notch ligand Jag1 (Fig. 2D). RV structures were detectable until PC39 (yellow arrow), and few s-shaped bodies (SSB) were still present at PC40-PC41 (white arrow). No JAG1 + signal was detectable in the nephron after PC41, in agreement with our histologic evaluation (Fig. 1) suggesting nephrogenesis was completed by PC40-PC41^22,23^. In summary, molecular analysis established that the naïve NPC population (SIX1 + /SIX2 + /WT1 + /ZO-1-) was exhausted by PC35-36 (P4-P5), with MET and nascent nephron formation completed by PC40-41 (~ P10-P11).

### 3D Renderings and multiplex in situ hybridization of Nephrogenic Niche show Evidence of arcades, but no evidence of lateral branch nephrogenesis

Three-dimensional (3D) rendering of confocal images is a powerful analytical tool enabling detailed morphological analyses the nephrogenic niche that would otherwise be missed. Thick section (1000 µm) were cleared and imaged along the Z axis (from the cortex towards the medulla) as previously described^18^. Due to the technical challenge of high non-specific background signal when using rabbit antibodies on thick sections of rabbit tissue, we could not visualize SIX1 + or SIX2 + staining  in the NPC. We therefore utilized conjugated antibody WT1 to represent the naïve NPC population as well as podocytes (see methods for further details).

**Figure 4:**
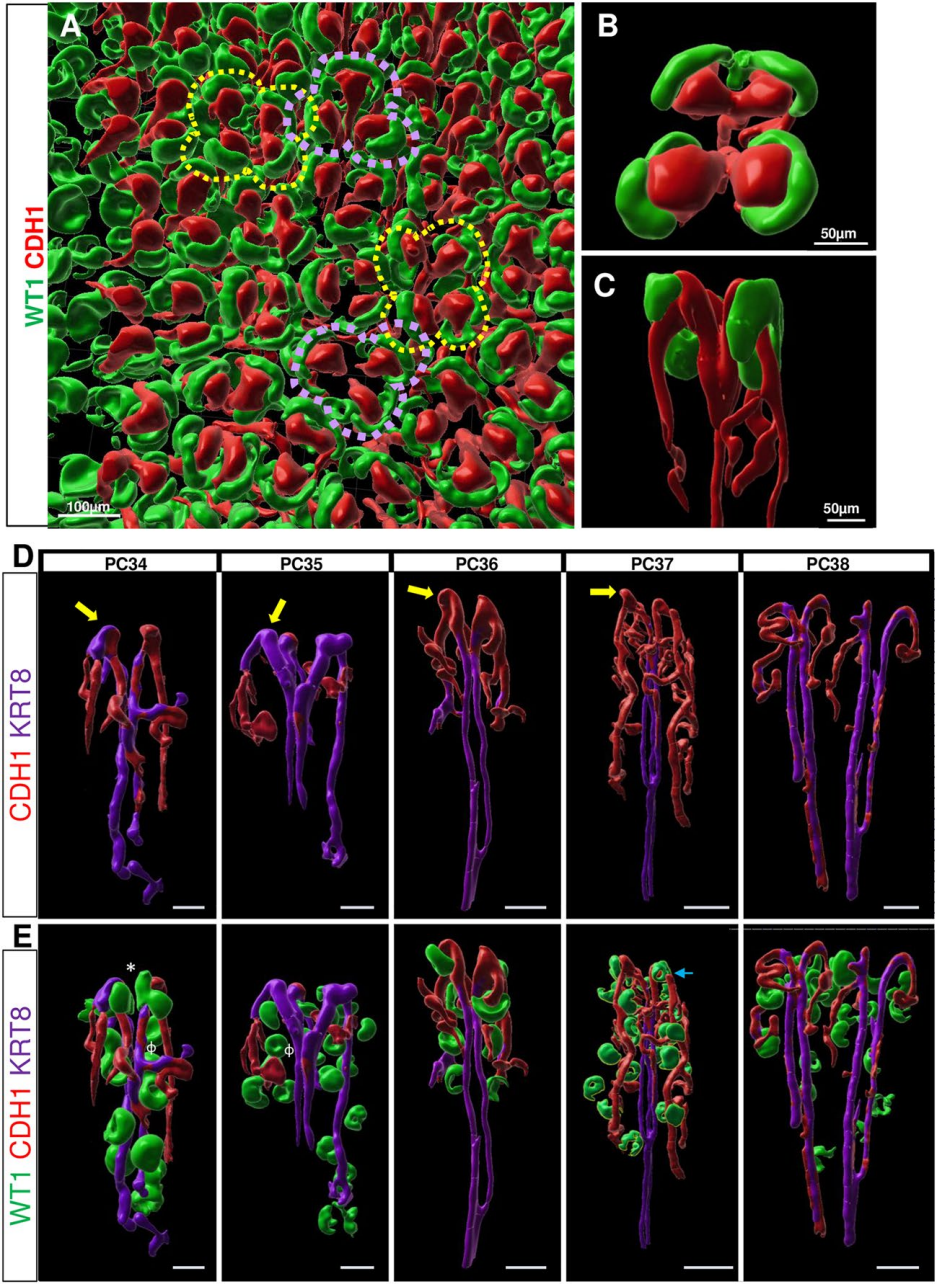
3D rendering of the rabbit nephrogenic niche shows evidence of rosette-like organization and arcades. 3D images reconstructed using Bitplane Imaris (**A**) Cortical view of the rabbit nephrogenesis demonstrates rosette-like cluster organization stained for WT1 (green) and CDH1 (red). Dashed lines drawn around rosette clusters represent the number of tips per niche cluster(purple = 3, yellow = 4). Isolation of one ureteric stalk demonstrating 4 niche tips from (**B**) cortical and (**C**) lateral view demonstrating long CDH1 + arcades. (**D**) Arcades are noted from PC34-PC38 with evidence of glomeruli (WT1) in close vicinity to chains. WT1 in progenitors is denoted by * while mature podocyte staining is identified by ɸ. By PC37, the WT1 + progenitor population has either migrated below the tip and shows evidence of lumen formation (blue arrow). UB horns identified by yellow arrow. Scale bar 100 microns.

Bird’s eye view of the rabbit kidney surface (looking at the X and Y axes) revealed rosette-like organization of the nephrogenic niches (Fig. 4A), as previously described in 23 week human^30^ and 129 day rhesus macaque^18^ kidneys. In all three species, the ureteric tip is bulbous and capped with asymmetric NPC. To further visualize the nephron structure, Z-stacks were assembled, rotated, and ureteric stalks were isolated and visualized along the Z axis (Fig. 4B,C). We identified elongated tubules with associated glomeruli extending below the ureteric bud (UB) tip, consistent with PBN arcades (Fig. 4D,E, Supplemental Fig. 2). Glomerular positions along the stalk were identified using WT1 and lie in close vicinity to the chains, although this antibody staining cannot visualize how the connecting segment of the newly formed nephron fuses to the chain of nephrons in the arcade. From PC34 to PC37, the UB tip above these arcades contained a horn-like protrusion interacting with an immature WT1 + NPC population (yellow arrow). The WT1 + cells wrap around the UB tip asymmetrically, and even display evidence of lumen formation by PC37 (blue arrow) as MET progresses.

**Figure 5:**
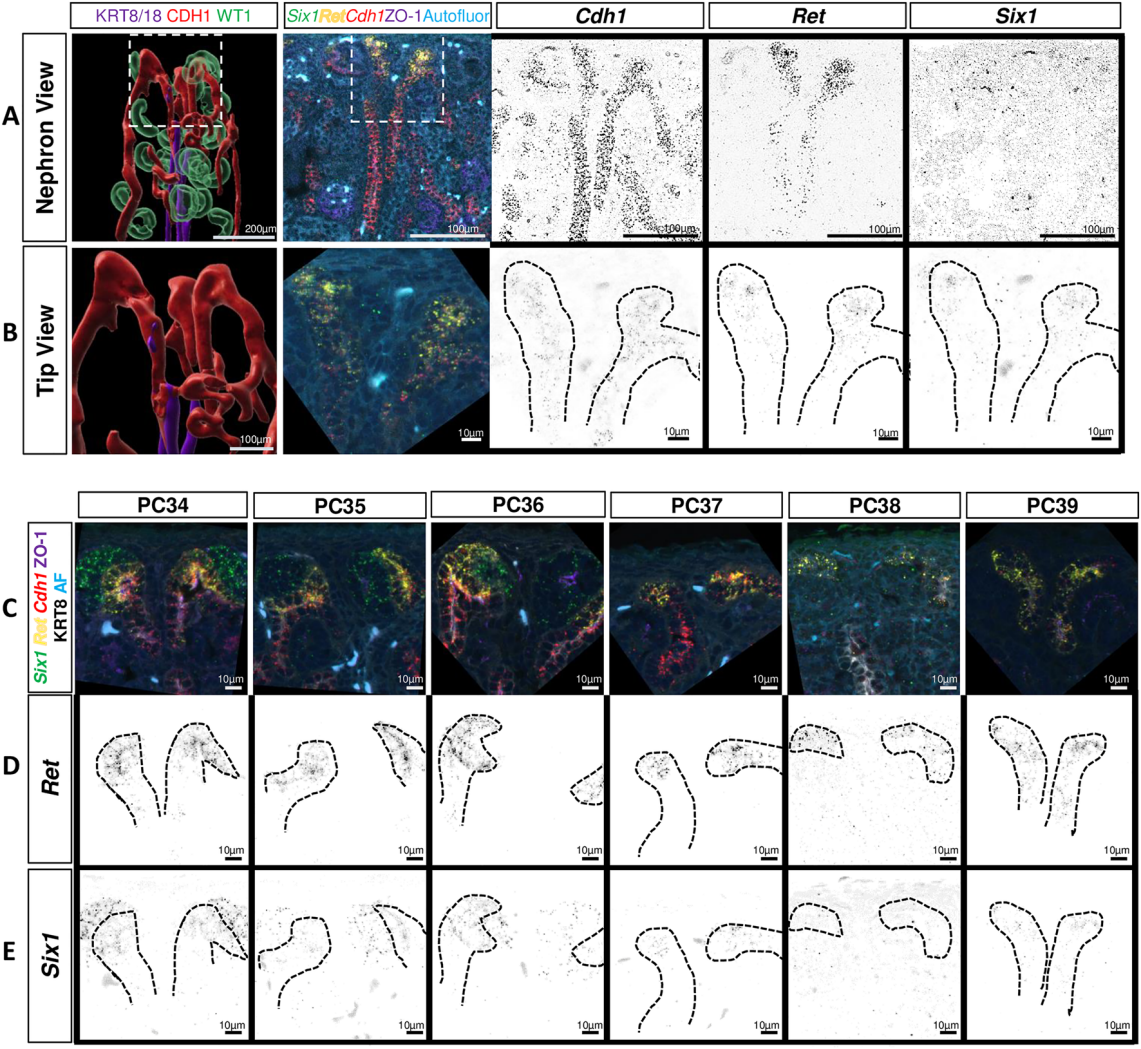
Multiplex In Situ Hybridization confirms early nephrogenesis cessation and ureteric bud/nephron progenitor interaction during arcading in the postnatal rabbit. (**A**) RNAScope was performed on PC37 rabbit kidney tissue. *Ret *+ UB horns visualized and project above the *Cdh1* + /*Ret*- arcades. (**B**) Inset from A, with depletion of a *Six1* + cap mesenchyme interacting with the UB horn. *Six1* + expression now visualized in the UB tip. (**C**) RNAScope was performed on postnatal rabbit kidney tissue from PC34 to PC39 with RNA probes for *Six1* (green), *Ret* (yellow), *Cdh1* (red), as well as antibodies to ZO-1 (purple, epithelized cells) (**D**, **E**) *Six1* + NPC are depleted after PC35, but *Six1* + staining remains in the UB tip at PC39. *Ret* + UB tips remain present through PC39.

To better characterize this distinct UB/NPC interaction and the arcades, we performed multiplex RNA in situ hybridization (ISH) (RNAScope©) on kidneys aged PC34 to PC39 (Fig. 5). We detected *Ret* expression in the UB horn and localized *Six1* transcripts in the nephrogenic niche. We used *Cdh1* to evaluate distal tubule epithelization. The UB horns were found to be *Ret* + */Cdh1* + , while the arcades were *Ret -/Cdh1* + and the UB was *Krt8/18* + (Fig. 5A,B). RNA expression of *Six1* was noted in the NPC until PC35 (P3-4), consistent with our immunofluorescent staining (Fig. 5C,E). Despite the early loss of *Six1* + signal in the cap mesenchyme, *Ret* signal persists in the UB tip until PC39 (P7-8), long after NPC exit from the niche (Fig. 5D).

UB tips remain *Ret* + as long as glial-derived growth factor (GDNF) is provided^31^. We thus performed ISH for *Gdnf* and found it to be strongly expressed during active nephrogenesis (PC34–PC36, P2–5), with faint stromal signal still visible by PC39 (P7-8) (supplemental Fig. 3). Unexpectedly, we also noted *Six1* + expression in the UB tip until PC39 (Fig. 5E, supplemental Fig. 4). To integrate these findings, we developed our nephrogenic scoring system, where a score of 5 equates to ongoing nephrogenesis while a score of zero lacks any evidence of ongoing nephrogenesis (Table 1). A nephrogenic score of 5 was achieved until PC35-36 (P3-5), with a score of zero in all but one sample by PC40 (P8-9). We conclude that active nephrogenesis defined by presence of naïve NPC persisted only until PC35-36 (P3-5), at which point no active niche tips with naïve NPCs were present, and no new nephrons formed after this short postnatal period.

## Discussion

Genetically tractable animal models are invaluable for molecular studies needed to uncover and validate the mechanisms controlling kidney development. The murine model is informative for many aspects of metanephric development but lacks the LBN phase of primate nephrogenesis^16^.

Although the spatial topography of the en masse differentiation of nephrons in the mouse on P2 may be superficially similar to early arcades, each nephron connects to the UB tip in a period too short for manipulation^17^. In this study, we defined the window of postnatal nephrogenesis in another rodent model, the rabbit. We find that the rabbit’s PBN window is shorter than previously thought^22^, but is significantly longer than the mouse. Like the mouse, there is no evidence for lateral branch nephrogenesis in the rabbit^15,18,19^. We identified many molecular similarities between the human and rabbit not seen in the mouse, further suggesting that the rabbit could serve as a model for human arcading. These similarities include rosette-like orientation of the asymmetric nephrogenic niche, primacy of SIX1 as a lasting marker of rabbit NPC, and the presence of elongated arcades^21^.

*Six1* is required in the mouse for maintenance of the early nephrogenic progenitors (E10.5)^27,32–34^, but is no longer detectable as soon as BpN begins. An equivalent role for *SIX1* in human (4–5 weeks gestation) or rabbit is unexplored. Not only is SIX1 detected throughout nephrogenesis in the rabbit and human, it persists after MET longer than SIX2^27,30^. In the mouse, forced expression of *Six1* under *Eya1* control failed to rescue the kidney of *Six2* null mice^35^. The rabbit is the first genetically tractable non-primate expressing SIX1 in the NPC population^27^ and thus may enable the study of *Six2* null mutant in *Six1*-expressing NPC and *Six1* null mutants in Six2-expressing NPC to map the non-redundant activities of these TFs.

Unlike the rabbit, the human and rhesus typically complete nephrogenesis prior to birth^36^. Preterm birth, however, is associated with increased risk of low nephron endowment, and increased risk of chronic kidney disease early in adult life^3,7,9,37–39^. Preterm infants must continue nephrogenesis postnatally. While any infant born before 37 weeks gestation is considered preterm, those less than 28 weeks are considered extremely preterm and at greatest risk^40^. The histologic findings point toward disrupted nephrogenesis, but the mechanistic reasons for these abnormal findings remain unknown^6,25^, hampering intervention efforts. With increasing success of resuscitation of human infants at the lowest limits of viability^41,42^, developing models of PBN in the neonatal environment will be clinically relevant.

McVary et al.^23^ evaluated nephrogenesis in the rabbit by performing unilateral ureteral obstruction of the kidney at term birth. They too observed attenuation of the nephrogenic zone by PC40, but reported continued increase in cortical glomeruli numbers reaching a plateau at PC48. While some may argue that nephrogenesis continued past PC40 in the rabbit, closer inspection of their study design revealed that the progressive increase in cortical glomerular counts from PC40 to PC48 was attributed to the progressive maturation of nephrogenic glomeruli (nascent/early nephrons) which the authors counted separately. When combining both counts, no new nephrons were added past PC40.

Even with the limitations of immunofluorescent imaging when using rabbit antibodies in a rabbit model, 3D modeling and multiplex RNA in situ hybridization methods establish that the rabbit as a non-primate model of arcading. Our molecular characterization of postnatal nephrogenesis identified a single *Ret* + UB tip protruding above the arcades and interacting with an asymmetric cluster of nephron-producing NPC (SIX1 + , Wt1 + , ZO1-) for several days. The finding of an active UB tip interacting with the naïve NPC is similar to human arcading and different from the mouse, where the differentiation of NPC en masse are all attached to the UB tip. In the rabbit and human, arcading seems to be an active process where a non-elongating UB maintains a nephron-producing niche for several days or weeks in a distinct post-branching process. The full length of the arcading period in the rabbit remains to be determined.

Importantly, our findings suggest that studies and/or manipulation of the postnatal nephrogenic niche in the rabbit should be completed during first 4–5 days of life (PC35–36), while studies aimed at early nephron formation and maturation can be continued up to the first 10–11 days of life (before PC40–41). With this knowledge, current rabbit experimental models of kidney development, and their translation to human health, need to be re-evaluated. Charlton et al^24^ study analyzed the impact of nephrotoxins during postnatal rabbit kidney development from P7–10 (~ PC36–39). Clearly an important contribution to pediatric nephrology, this study targeted the window of nascent nephron maturation, not the nephron progenitor niche. It demonstrates the negative impact of nephrotoxins on the early epithelial nephrons, but the vulnerability of the NPC to nephrotoxins remains unknown. Interestingly, McVary et al.^23^ studied the impact of unilateral ureteral ligation on nephron development, and whether timing of ligation at PC22 (in-utero) versus PC31 (term) impacted nephron number. They reported that in order to produce a similar deficit in nephron number, 6 days of obstruction in utero (PC22–PC28) was required versus 2 days of obstruction at PC31. Since they report that the nephrogenic zone was only minimally impacted by the obstruction, the results suggest that postnatal arcading may be more sensitive to injury, or that injury to maturing nephrons has the most negative impact on long-term nephron number rather than on the nephrons still being formed.

Although the rabbit does not display LBN, it is the first non-primate model to display SIX1 dependent nephrogenesis with a postnatal window of PBN arcading amenable to manipulation. We were unable to visualize all segments of the nephron due to limitations in non-rabbit antibody availability limiting multiplexing and 3D rendering, but if the rabbit becomes a more widely used model for the study of nephrotoxicity and other neonatal exposures, antibodies will be raised in other hosts. We were able to visualize active nephron-inducing UB tips, with elongating CDH1 + chains extending below with close association to the WT1 + glomeruli. We could not visualize the fusion of connecting segment from one nephron into the distal segment of another typical of the arcade architecture, and have to rely on other studies^21^ to bridge this gap. With better reagents, the rabbit offers an opportunity to characterize in molecular detail how the connecting segment of the newly formed nephron fuses to the chain of nephrons below, and not the duct. Future efforts could also focus on molecularly defining the signaling pathways and ligand-receptor interactions between the arcading UB tip and the BpN branching tip, respectively, and the NPC during BpN and PBN. An unexpected finding in the rabbit is that before cessation, Six*1* + transcripts accumulate in the UB tip, maintained by stromal GDNF. We are unable to identify SIX1 protein, and while our efforts were confounded by high background, differential translation^43^ of *Six1* mRNA is also a likely explanation. Whether Six*1* + accumulation in the UB tip is reflected in SIX1-dependent gene expression, and whether *Six1* is transiently expressed in the UB tip in the transition from arcading to LBN, remains to be explored.

## Methods

### Rabbits

Timed-pregnant New Zealand rabbits were purchased from Charles River and cared for per Cincinnati Children’s institutionally approved IACUC protocol 2016–0032 and 2021–0067, and methods were carried out in accordance with relevant guidelines and regulations. All methods are reported in accordance with ARRIVE guidelines. A total of 35 rabbit kits were obtained from four timed-pregnant females. Kidneys were harvested via transcardiac perfusion using PBS and either 4% PFA or 10% formalin (RNAScope samples). One kidney was processed and embedded in paraffin by the CCHMC pathology core, while the other kidney was stored in PBS with 0.01% sodium azide for clearing and thick section immunostaining.

### Hematoxylin and eosin (H&E) staining and processing

H&E was performed with the assistance of the Cincinnati Children’s pathology core. Five-micron sections of paraffin embedded tissue was stained using the Ventana Symphony automated H&E slide stainer and scanned using the Nikon Ti2 Upright Widefield Microscope at 20 ×  objective. Images were processed using NIS Elements software. The width of the nephrogenic zone was determined by the area in the outer renal cortex exhibiting developing nephrons in the form of s-shaped or comma-shaped bodies. Distinct regions per kidney were chosen with 6–8 separate measurements per region.

### Immunofluorescence

Five-micron sections of paraffin embedded tissue were used for Immunofluorescence. Immunofluorescence was performed as previously described^18^ with the following changes. The slides were blocked and permeabilized in PBS with 6% bovine serum albumin (BSA), 0.1% triton, 0.01% sodium azide, with 5% normal donkey serum (NDS) for 1 h at RT, followed by wash × 1 in PBS for 10 min. To overcome the non-specific staining of a rabbit antibody on rabbit tissue, we performed a 5 min incubation using Scytek Superblock. After washing four times in PBS for 5–10 min at RT, we then performed an incubation in Scytek rabbit-to-rabbit blocking reagent for 1 h at RT followed by PBS wash × 4 for 10 min each before adding primary antibody. For all immunofluorescent studies, four animals were analyzed at PC31 to CP33, three animals per time point were analyzed from PC34 to PC40 and one animal was analyzed at PC41 and PC42, respectively. Due to poor fixation in the tissue in one animal at PC37, only two of the three animals from PC37 were able to be analyzed.

### Thick tissue clearing and staining

A 1000 µm cortical sections of rabbit kidneys were cleared as previously described^18^ with the following exception: 25% N,N,N′,N′-Tetrakis(2-Hydroxypropyl)ethylenediamine (Quadrol) was not used as this diminished WT1 signal. One representative kidney sample from one animal from PC33 to PC38 was analyzed in this study.

### Fluorescent microscopy

Cleared thick tissue sections were mounted in a falcon tissue culture dish within a press-to-seal silicone 1 mm thick adhesive silicone isolator. The tissue is submerged in RIMS and covered with WillCo-dish Glass bottom dish. Three dimensional (3D) images were obtained using a Nikon AXR upright Microscope using the 10 × glycerol objective. 1000-mm z-stacks were obtained at 512 × 512 pixels using 2.6 mm step size at 2.51 mm/px. Paraffin embedded section images were obtained on the Nikon A1 Inverted Confocal Microscope and Nikon inverted AXR using the 20 × objective.

### In situ hybridization

In situ hybridization was performed using the Multiplex Fluorescent V2 Assay (Advanced CellDiagnostics, Inc.) according to the manufacturer’s protocol. Positive controls used were rabbit PPIB (medium abundance) and POL2RA (low abundance). The negative bacterial control (DapB) was used on all tissue samples. Probes used included rabbit *Six1*, *Six2*, *Ret*, *Cdh1*, and *Gdnf*. Images were obtained on the Nikon AXR inverted microscope and Ti2 upright microscope at 20 × objective. One animal at each respective post conceptual age with two representative sections was analyzed in this part of the study.

### Image processing and analysis

NIS-Elements 5.3.00 was used for image processing, measurement of nephrogenic zone width and conversion of ND2 files to TIF files. 3D tissue z-stack Images were processed using Bitplane Imaris 9.5.1. Surfaces were created using surface rendering, described as follows: After opening the image in Imaris, “add new surface” is selected for each immunofluorescence channel, and background subtraction was used for thresholding the surface, with further thresholding cutoffs determined by immunofluorescent staining for the entire z-stack. Individual niche surfaces were then isolated using the select and “duplicate” function within surface rendering to create a duplicate copy of the individual niche without removing the original surface. Statistical Analyses were performed using GraphPad Prism v9. Mean ± SD and linear regression was determined for the nephrogenic zone width.

### Antibodies

Primary antibodies used include rabbit anti-Six2 (Proteintech, 11,562-1-AP, 1:1000), rabbit anti-Six1 (Cell Signaling, #12,891, 1:500), guinea pig anti-cytokeratin 8 + 18 (Abcam, AB194130, 1:500 and Progen, GP-KP, 1:500), mouse anti-zo-1 (Invitrogen, 339,100, 1:400), mouse anti-E-cadherin (BD biosciences, 618082, 1:400), mouse anti-WT1 (Santa Cruz, sc-7385, 1:50) JAG1 (Santa Cruz, sc-8303, 1:100).

### Supplementary Information

Supplementary Information.

## Data Availability

All images and measurement data will be made availability in supplementary files.
