# Supplementary material for: Characterizing post-branching nephrogenesis in the neonatal rabbit
Source: Sci Rep. 2023 Nov 6;13:19234. doi: 10.1038/s41598-023-46624-9 (PMC10628296; doi:10.1038/s41598-023-46624-9)
Supplement: Supplementary file 1 — Supplementary Information. [file 41598_2023_46624_MOESM1_ESM.pdf]

Supplemental Material Table of Contents

1. Supplemental Methods
2. Supplemental Figures
3. Supplemental Figure legends

1. Supplemental Methods:

Rabbit Protocol and harvesting kidneys

Timed-pregnant New Zealand rabbits were purchased from Charles River and cared for per IUCAC protocol 2016-0032 and 2021-0067. Kidneys were harvested as follows: Each kit was given anesthetic overdose of pentobarbital (65mg/ml concentration,0.08ml-0.4ml based on weight). Kits were assessed with toe pinching and next steps not proceeded unless unresponsive to noxious stimuli. After securing the rabbit in the supine position, we made an incision along the thoracic midline from xiphoid process to clavicle. We then reflected the two flaps of skin laterally to expose the thoracic field. We then cut through the thoracic muscles and ribcage along the midline. Next, we separated the diaphragm from the chest wall on both sides, and then pin the reflected ribcage laterally to expose the heart and thoracic organs. We then secured the heart with blunt forceps and make an incision (1-2mm minimum) into the left ventricle. Immediately after, we inserted a needle (20 to 26 gage) with 20 to 35cc syringe filled with PBS and secured with hemostat. We then will cut the right atrium with scissors and at the first sign of blood flow we manually infused PBS. We continued to perfuse until the fluid exiting the right atrium was clear (15ml-200ml based on size). We followed this by infusing 4% PFA (10-80ml). Kidneys were then harvested and placed in 4%PFA on ice for further analysis. Kidneys were further fixed in 4% PFA by rocking for 1 hour at room temperature (RT) for PC31-39, or at 4ºC overnight for PC40-49 due to larger size. After fixation, kidneys were cut in half. One kidney was processed and embedded in paraffin by the CCHMC pathology core, while the other kidney was stored in PBS with 0.01% sodium azide for clearing and thick section immunostaining. Kidneys were collected from PC34 to PC37). Any kidney used for RNASCope was fixed in 10% formalin rather than PFA.

2. Supplemental Figures

Figure S1

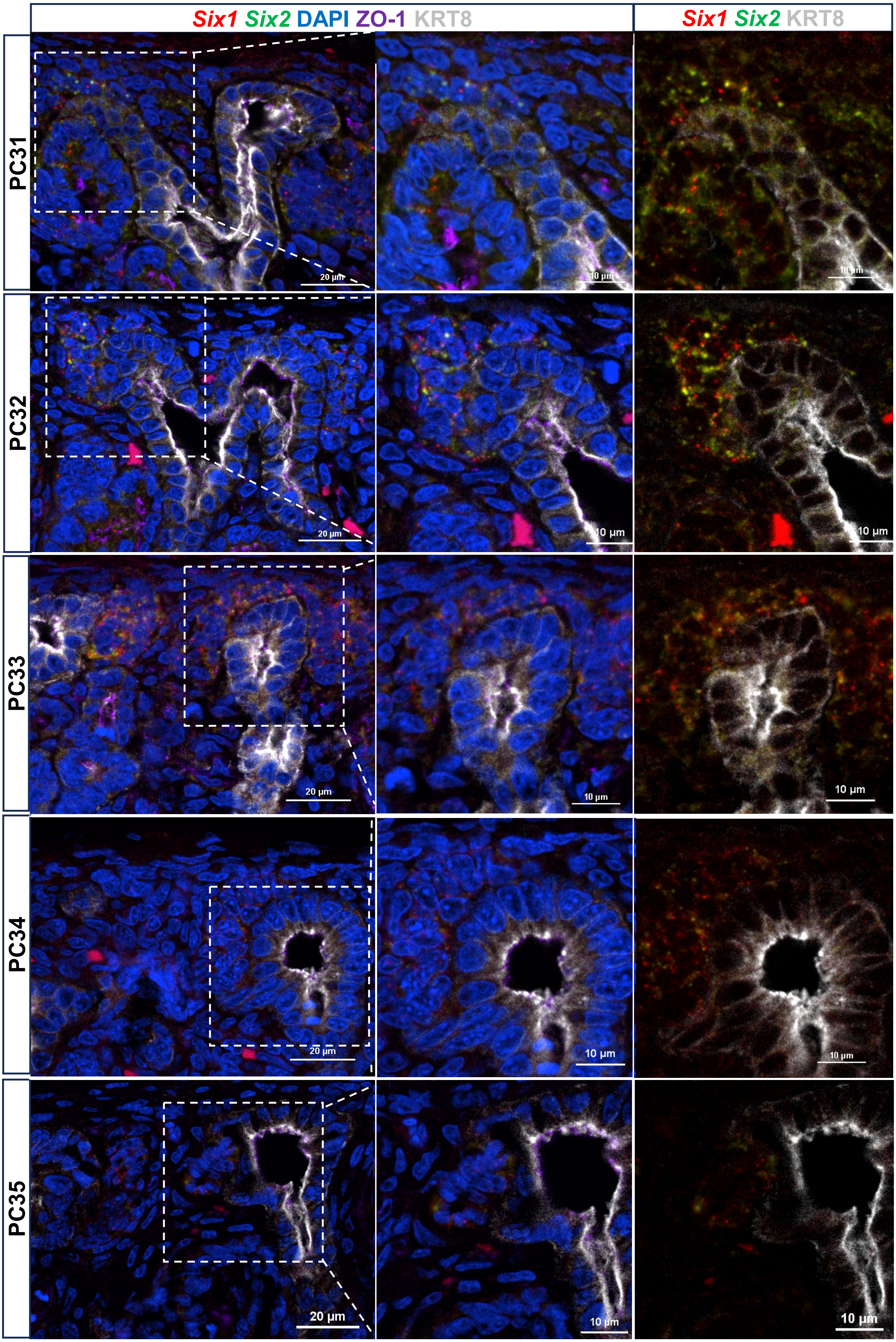

Six1 Six2 DAPI ZO-1 KRT8
Six1 Six2 KRT8
PC31
PC32
PC33
PC34
PC35
20 µm
10 µm

Figure S2

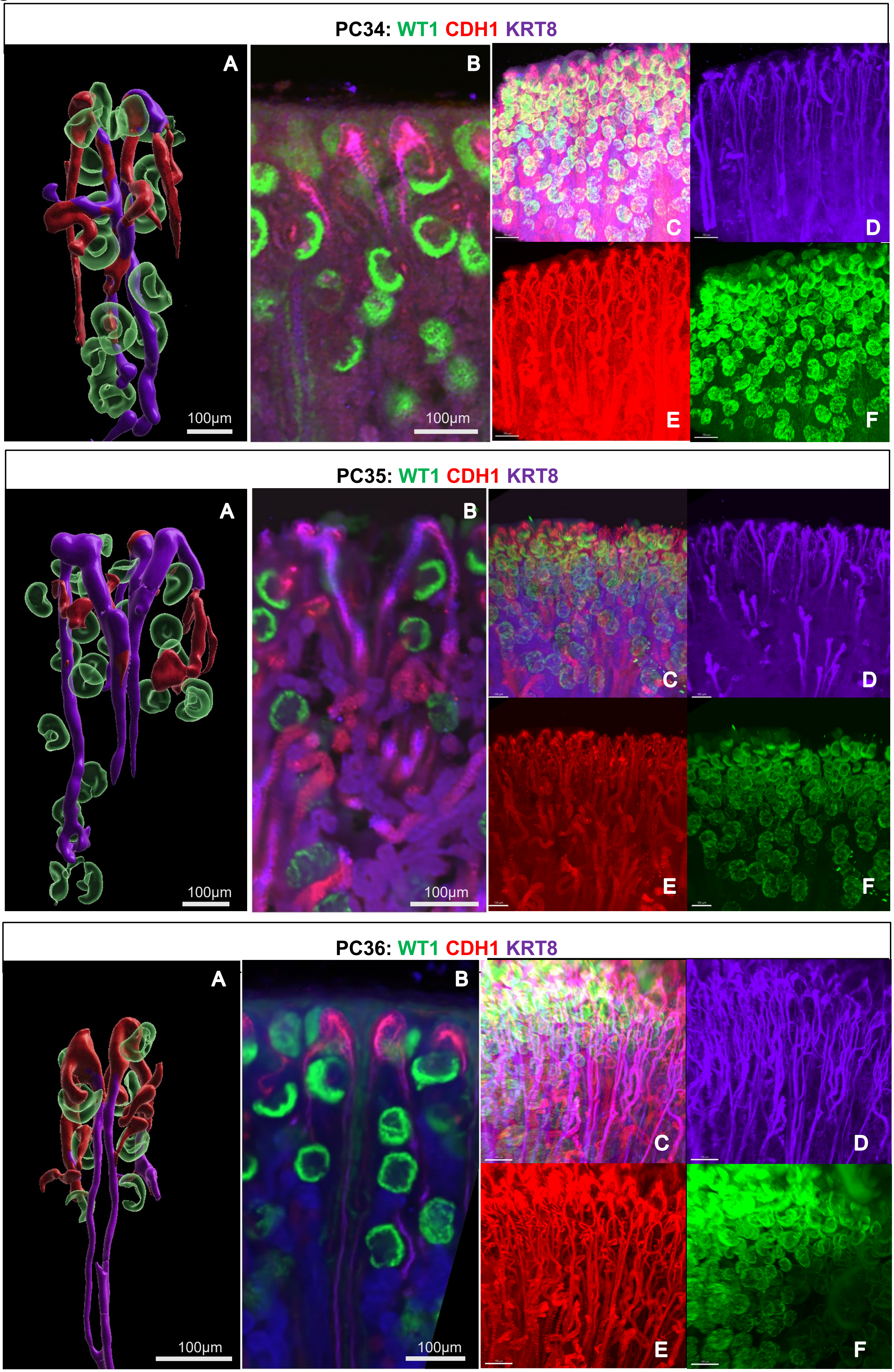

PC34: WT1 CDH1 KRT8
A
100µm
B
100µm
C
D
E
F
PC35: WT1 CDH1 KRT8
A
100µm
B
100µm
C
D
E
F
PC36: WT1 CDH1 KRT8
A
100µm
B
100µm
C
D
E
F

Figure S2 (cont)

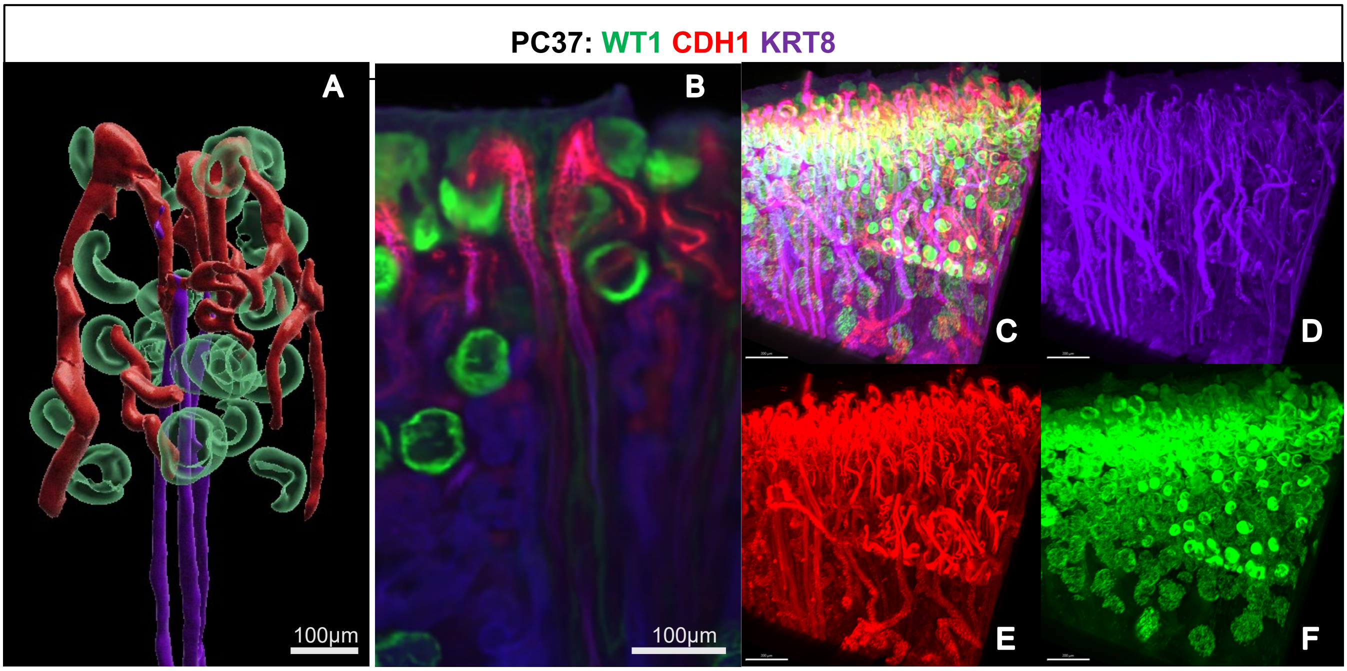

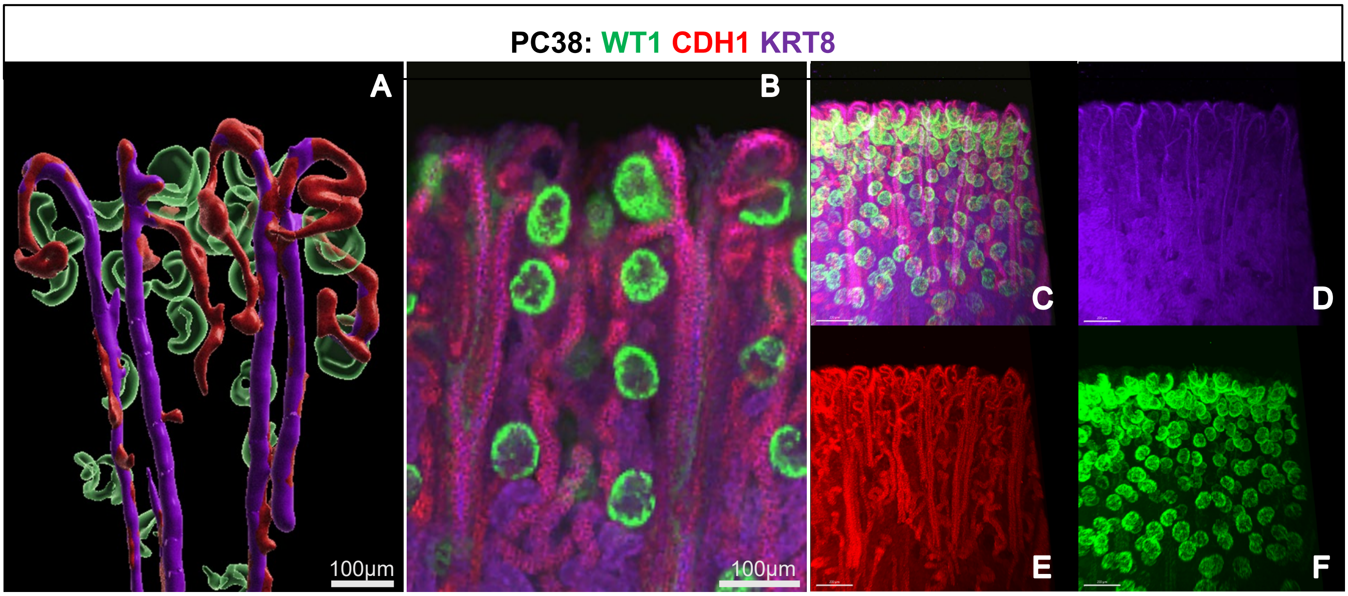

Figure S3

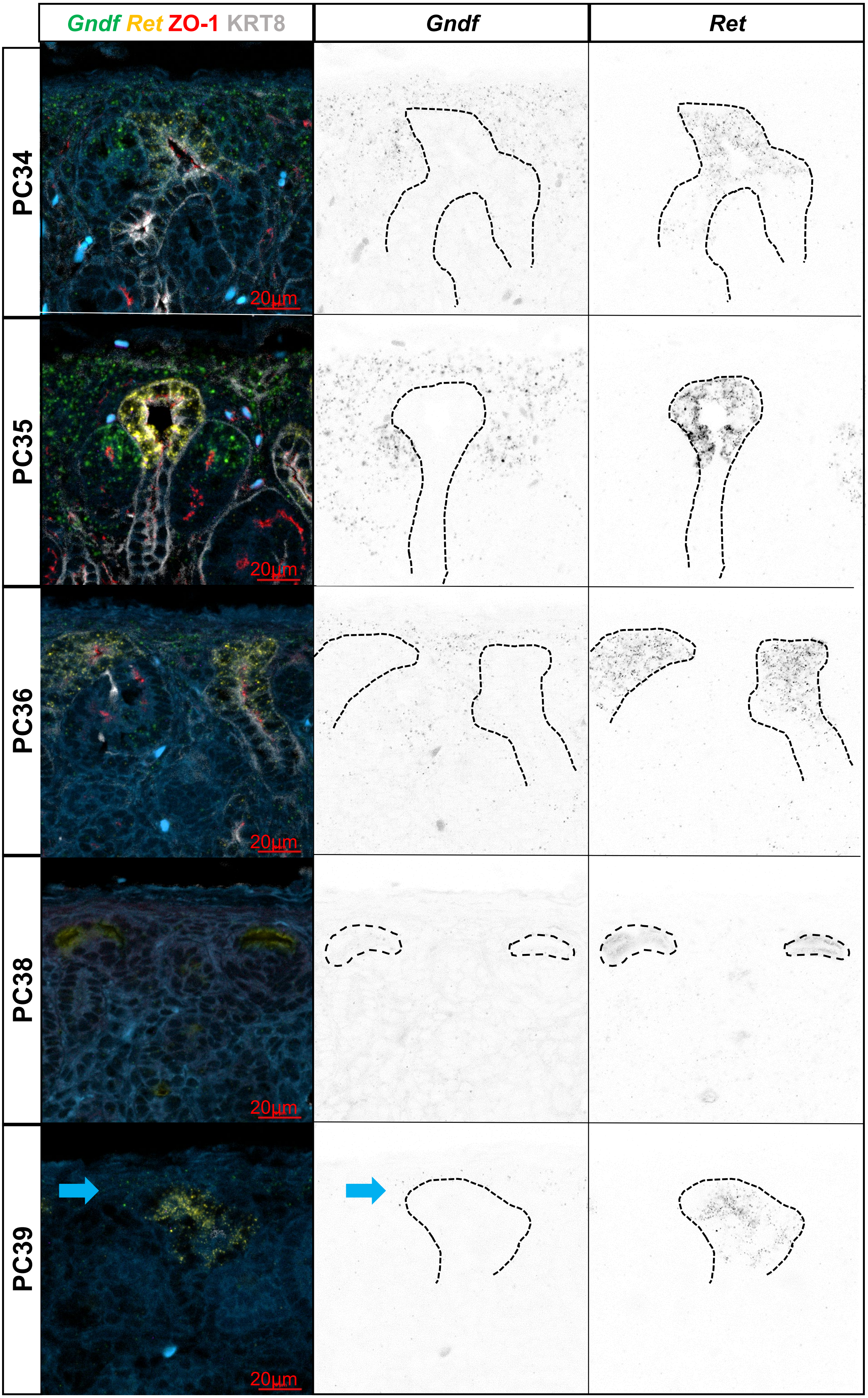

Gndf *Ret* ZO-1 KRT8
Gndf
Ret
PC34
PC35
PC36
PC38
PC39
20µm
20µm
20µm
20µm
20µm

Figure S4

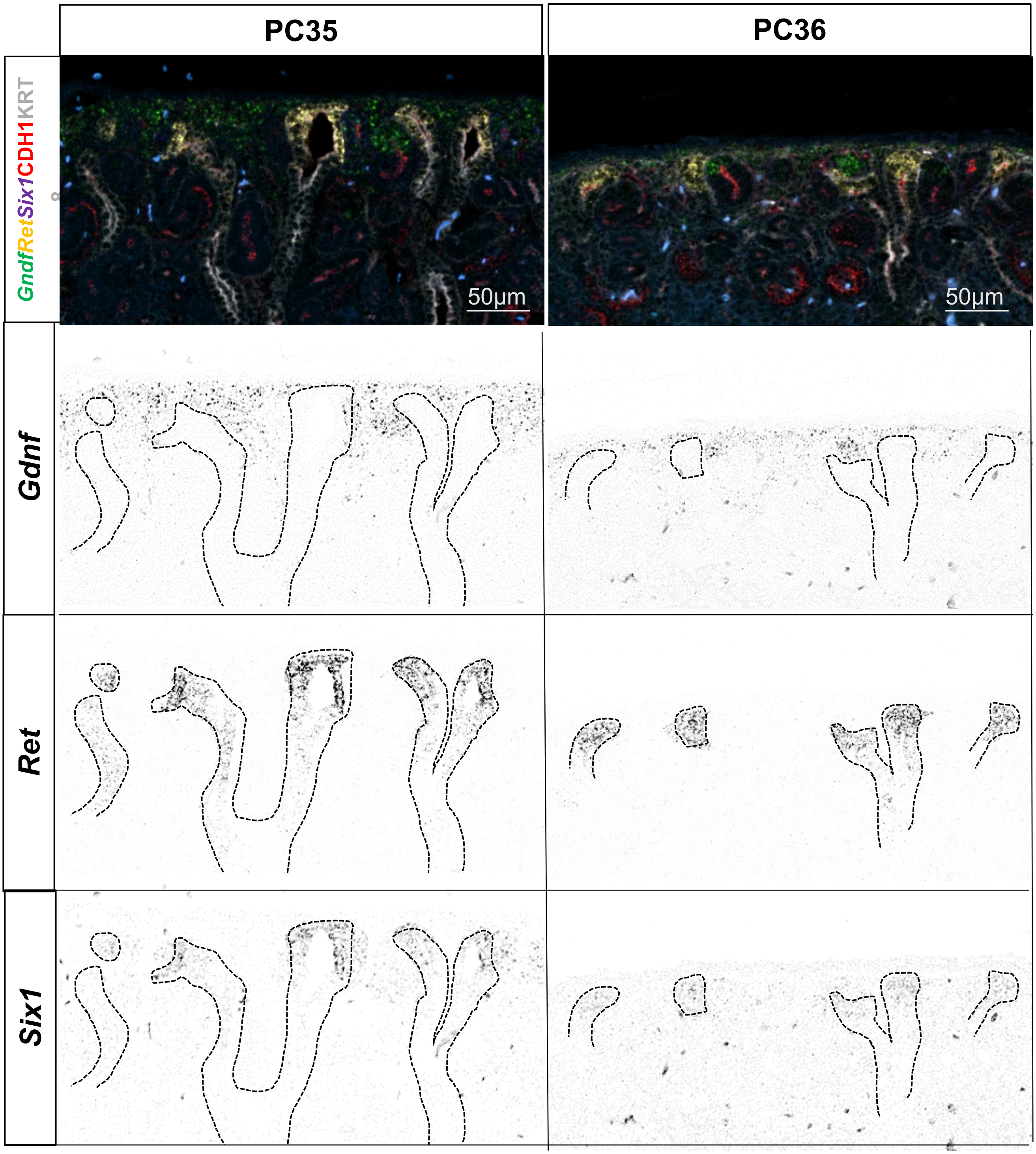

3. Supplemental Figure Legends

Supplemental Figure 1: *Six1*+ and *Six2* are co-expressed in the nephrogenic niche. 100x magnification displays both *Six1* (red) and *Six2* (green) transcripts within the same cell population interacting with the UB tip from PC31 to PC34.

Supplemental Figure 2: Comparison of 2D and 3D imaging of rabbit niche. 3D surface renderings (A) of individual niche using Bitplane Imaris, representative 2D slice from z-stack (B) of the 3D rendered niche, and (C) and composite overview of full z-stack of thick section in Imaris, as well as individual channels expressing (D)KRT8, (E) CDH1, and (F) WT1 in Imaris for PC33 through 38.

Supplemental Figure 3: *Gndf* is strongly expressed during active nephrogenesis. *Gndf* expression (green) is strongly expressed throughout the niche from PC34-36. Although poor in situ expression noted on PC38 due to tissue quality, faint *Gdnf* is still noted throughout the stroma at PC39 (blue arrow). *Ret* expression persists at PC39. 40x objective.

Supplemental Figure 4: Continued *Six1*+ expression noted in UB tip. Although the NPC have exited the niche, there is persistent *Six1*+ expression in the UB tip co-localizing with Ret expression using RNAScope RNA multiplex in situ hybridization. This finding is only seen after *Six1*+ progenitor exit from the NPC niche.
